# Covid-19 variants of concern and pregnancy

**DOI:** 10.1136/bmjmed-2022-000151

**Published:** 2022-03-02

**Authors:** Sarah J Stock, Clea Harmer, Clara Calvert

**Affiliations:** 1 University of Edinburgh Usher Institute of Population Health Sciences and Informatics, Edinburgh, UK; 2 Sands, London, UK

**Keywords:** pregnancy complications, COVID-19

A shifting landscape of covid-19 risk for pregnant women

If pregnant women are infected with SARS-CoV-2, they are more likely than non-pregnant women to have severe covid-19, with higher rates of hospital admission, higher rates of admission to intensive care, and an increased need for respiratory support.^1^ Covid-19 also increases the risks of pregnancy complications, including preterm birth (birth before 37 weeks' gestation), stillbirth, and newborn death.^1^ In short, covid-19 carries additional risks in pregnancy, which has implications for both women and their caregivers, and raises the question of how best to reduce these risks.

To date, most data around the risks of covid-19 in pregnancy comes from early in the pandemic, when wildtype SARS-CoV-2 predominated. In a linked *BMJ Medicine* research paper (doi:10.1136/bmjmed-2021-000053), Vousden and colleagues^2^ aimed to examine the impact of two variants of concern of SARS-CoV-2, alpha and delta, on the severity of maternal infection and perinatal outcomes when compared with the wildtype virus.^2^ The study was carried out through the UK Obstetric Surveillance System, which enables robust data collection from all NHS consultant led maternity units in the UK where pregnant women with covid-19 receive care.^3^ The study by Vousden and colleagues included over 4000 pregnant women or women who had recently given birth, and who were admitted to hospital with confirmed SARS-CoV-2 infection and symptoms of SARS-CoV-2 infection.

The study by Vousden and colleagues provides unique insights into the impact of SARS-CoV-2 variants on the severity of infection in pregnant women in the UK. As seen in non-pregnant populations,^4 5^ the alpha and delta variants have been associated with more severe disease in pregnancy than the wildtype. An increasing proportion of women admitted with covid-19 related symptoms had moderate to severe infection over the periods when the wildtype, alpha variant, and delta variant predominated; ranging from 24.5% in wildtype dominant periods to 42.8% in delta dominant periods. After adjustment for potential confounding factors, the risk of admission to intensive care was higher in both alpha and delta periods than in the wildtype period.

These observational data have understandable limitations—especially regarding the absence of viral sequencing data, when the authors had to use time periods as a proxy for the variant of concern. However, they had a strong justification to underpin the cut-off dates for the different time periods, and overall this provides convincing evidence of the increasing severity of maternal infection with the alpha and delta variants.

The findings reported on the association between SARS-COV-2 variants and perinatal outcomes are more circumspect. Although the risk of admission to a neonatal unit in relation to mothers admitted during the alpha dominance period was higher than those admitted during the wildtype period, no evidence indicated a difference between these periods for stillbirth and data on neonatal deaths were insufficient. These data reflect inherent challenges in studies of perinatal outcomes. Firstly, to accurately estimate pregnancy complications, a sufficient number of pregnancies need to have completed. Thus, a time lag exists before the effects of variants on pregnancy outcomes and babies can be examined. In the linked study, perinatal analysis was limited because during the delta period, only two thirds of women had completed their pregnancy. Secondly, a sufficient number of eligible people would be needed to accurately estimate risks, because small numbers result in uncertainty and potentially misleading rates. A tension exists between the timely release of data and prompt identification of signals of concern, and the allowance of sufficient data to accumulate to allow more robust analysis to avoid creating unnecessary anxiety for parents. Further data are required to more fully understand the impact of different variants on perinatal outcomes.

The study by Vousden and colleagues was not designed to determine the effect of vaccination on severe covid-19 in pregnancy, although it did report on this important aspect of care. In the period when vaccination status was examined, only 3% of pregnant women admitted to hospital with covid-19 related symptoms were vaccinated. Despite being based on only a small number of pregnant women with the most severe outcomes of SARS-CoV-2, these data reflect findings from other studies indicating that covid-19 vaccination is protective against covid-19 complications in pregnancy.^6^

The linked study also provides insights regarding treatment of covid-19, with considerable undertreatment of severe covid-19 in pregnancy observed. For example, by the start of the delta period (around June 2021), evidence based guidance recommending corticosteroids for the treatment of pregnant women with covid-19 who require oxygen was well established, yet Vousden and colleagues found less than 30% of pregnant women who were admitted to intensive care with covid-19 during the delta period received steroids. By contrast, rates of treatment in non-pregnant populations seem much higher, even considering potentially different thresholds for admission and treatment. For example, a recent UK study found that in June 2021, 80% of all intubated patients in intensive care with symptoms of covid-19 received steroids.^7^ These levels of steroid use demonstrate that a culture of caution regarding drug treatments in pregnancy persists, even when evidence clearly indicates that undertreatment is more dangerous than any putative detrimental effects of the drug itself.

Consistent messaging is required to counteract pervasive misinformation about both treatment of and vaccination against covid-19 in pregnancy. The resulting confusion for women results in anxiety and affects their ability to make informed decisions. Caregivers also need to feel confident in recommending effective treatments, which requires good quality evidence relevant to pregnancy. The landmark RECOVERY trial included pregnant women,^8^ and this inclusion should be the standard rather than the exception. Further work is needed to ensure pregnant populations are offered trial participation and are not excluded from the implementation of evidence based treatments that can be lifesaving.

But messaging alone will not increase vaccination rates in many settings, owing to the continued inequity of vaccine access globally as well as the inconsistent policies regarding vaccination in pregnancy. Nevertheless, signs of a shift in global policies have been encouraging. For example, between June 2021 and January 2022, the number of countries recommending covid-19 vaccination to all or some pregnant women increased from 12 to 94.^9^ Some settings have shown signs of increased vaccine uptake in pregnancy in recent months,^10^ although Scottish data show that lower levels persist in black, Caribbean, or African women when compared with women from other ethnic groups.^10^

Continuing to ensure that all pregnant women are offered and have access to vaccination is particularly important given the continued high levels of infection with the newest variant of concern, omicron. Despite evidence from non-pregnant populations that omicron might be less severe, it should not be considered mild, especially in unvaccinated individuals,^11^ and it certainly cannot be assumed that the new variant's effects in pregnancy will be the same as in the general population. Therefore, further research to build on Vousden and colleagues' findings is urgently needed, to ensure that pregnant women and healthcare providers have access to essential evidence on the specific effects in pregnancy to inform decision making. Although robust, population level data will take time to generate, such as those from the UK Obstetric Surveillance System, they remain critical to mapping the shifting landscape of covid-19 risk to pregnant women and their babies.
